# Otology

**Published:** 1907-04-13

**Authors:** 


### OTOLOGY.

### ACUTE OTITIS MEDIA.

Although affecting adults, this condition is particularly common in infants. In a child that is ill it is as essential to examine the ear as it is to examine the throat, as the following case illustrates:—

A well-fed child of 13 months was taken ill suddenly one evening, crying all night with pain. The temperature was 103. There was no sore throat. He was crying and fretful during the day, and wakeful the following night. The temperature remained between 103 and 104. There were no physical signs in the chest or abdomen, and the bones and joints were free. There were no digestive troubles. Influenza was prevalent at the time, and from this it was thought the child was suffering, especially as he had had a trifling cold in the head. On the fourth day the ears were examined, and there was no discharge.

Next day, that is the fifth, there was a sudden appearance of muco-pus in the left meatus, with some relief of pain, but the temperature still remained raised, and on the following night the child was as restless and wakeful as before. It was thought that the right ear was accounting for these symptoms. There seemed to be some relief afforded by hot applications to that side of the head. On the sixth day the ears were examined again. On the left side, after removal of thick stringy muco-pus by gently syringing with hot alkaline lotion and drying the meatus, the tympanic membrane was seen to be bulging and to have lost lustre; it was not injected, but was white as if sodden and œdematous. There was a pin-hole perforation through which a bead of muco-pus was escaping. On the other side there was no discharge, but the tympanic membrane was bulging, had lost lustre, and was uniformly red. There was no perforation. During examination the child remained quiet. The temperature was 103.

#### Operation.

Chloroform was given, and the left tympanic membrane was completely incised across, with a fine myringotome. With a freshly sterilised instrument the right membrane was also completely incised from above downwards. The fluid in the right middle ear was collected in a pipette, and was found to contain pyogenic streptococci which grew in cultivation in large numbers.

#### Treatment.

Both ears were syringed with alkaline boracic lotion, and in the meatus was laid a narrow strip of cyanide ribbon gauze, quite loosely, and reaching down to the incised membrane. In a week both membranes healed.

#### The Examination of an Infant's Ear.

This is not always an easy thing to do. A good light is essential. The head mirror should have a short focal length, not more than 9 inches. Cerumen is seldom present, but the meatus is often narrow and curved, and fine downy hair may make it difficult to obtain a good view. Use the largest speculum which can be employed. The light should be directed along the roof and posterior wall of the meatus, while the pinna is gradually drawn upwards and backwards to straighten the canal until the tympanic region comes into view.

#### The Normal Membrane.

The most important landmark of the normal membrane is the processus brevis of the malleus, which forms a little white spot near the upper and anterior part. From this white spot downwards and slightly backwards should be traced the handle of the malleus, as a short and nearly straight streak. Often a minute vessel can be traced down the handle of the malleus. Near the centre of the membrane the malleus becomes lost to view. Below, and generally a little in front, of the lower end is seen a lustrous triangle of reflected light. The rest of the membrane is less lustrous. It should be possible to make out the attachments of the membrane at its margin to the tympanic ring. This is more readily distinguished in front and below, while posteriorly it is not easy to say where the meatal wall ends and the tympanic membrane begins.

#### Inflammation and its Effects.

In acute inflammation of the tympanic membrane there is loss of lustre, and the whole field is injected red, so that none of the above-mentioned landmarks are visible except in the earliest stages. If inflammatory products accumulate in the tympanum the membrane is bulged outwards. In some cases the bulge may resemble a polypus in the meatus. More often it forms a red elevation, which appears to be partly on the posterior wall and partly in the tympanic region. In reality it is the posterior half of the membrane itself.

#### Treatment.

The first question to consider in such a case is, Should every bulging membrane be incised. In the case of pain, deafness, and pyrexia a very injected and bulging membrane should be freely incised. If there is deafness without much pain and no pyrexia, wait for, say, 12 hours and note the state of affairs. If there is still no rise in temperature, and the pain is subsiding and the bulging does not appear to be increasing, continue to treat by nasal and pharyngeal sprays to keep the Eustachian openings free from accumulation of discharge. A blood-count, to ascertain the number of leucocytes, should be made in difficult cases. If, however, pain returns or bulging increases, it is better practice to incise and drain freely than to wait for perforation to take place, or for the chance of its clearing up. If the membrane has already ulcerated and given way through a small perforation but is still bulging, it is better to incise freely than to leave alone. Otherwise the condition is very apt to become chronic, and will lead to permanent impairment of hearing.

